# SARS-CoV-2 infection in the context of Kawasaki disease and multisystem inflammatory syndrome in children

**DOI:** 10.1007/s00430-022-00756-3

**Published:** 2022-11-17

**Authors:** Barbara Anna Folga, Corrinna Jade Karpenko, Bogna Grygiel-Górniak

**Affiliations:** Department of Rheumatology, Rehabilitation and Internal Diseases, Poznan University of Medical Science, Poznań, Poland

**Keywords:** MIS-C, Kawasaki disease, SARS-CoV-2, COVID-19, PIMS

## Abstract

Recent studies have noted an increasing number of Kawasaki-like cases in the pediatric population following severe acute respiratory syndrome coronavirus 2 (SARS-CoV-2) infection. In the literature, the condition is described as multiple inflammatory syndrome in children (MIS-C) or pediatric inflammatory syndrome (PIMS). A similar clinical course of Kawasaki disease (KD) and MIS-C causes difficulties in distinguishing between both conditions. However, the differential diagnosis is crucial since patients with MIS-C can present severe symptoms (myocardial dysfunction, fever, mucocutaneous symptoms) and require more extensive monitoring during treatment than children diagnosed with KD. Along with assessing epidemiological and genetic factors, it is imperative to estimate the risk of developing MIS-C in KD patients with confirmed SARS-CoV-2 infection. Genetic predispositions, such as the *ITPKC* gene polymorphism in KD, ACE deletion (D) polymorphism in SARS-CoV-2, and inborn errors of immunity (IEIs) in MIS-C affect the regulation of immune system complex clearances and cellular adaptations. The virus has a tropism for both vascular and respiratory cells, which further causes additional symptoms necessitating standard therapy with antithrombotic treatment. The diagnostic criteria for KD, MIS-C, and SARS-CoV-2 help differentiate each condition and optimize treatment strategies. Unfortunately, long-term outcomes in KD patients who develop MIS-C due to SARS-CoV-2 infection have been inadequately documented due to the timing of the pandemic, further displaying the need for longitudinal studies in these patients. This review underlines the differences in diagnosis and treatment of KD and MIS-C. Overall, children with KD may develop MIS-C in the setting of SARS-CoV-2 infection, but further research is needed to outline specific etiologies, prognostic factors, and diagnoses.

## Introduction

Since the first case of the severe acute respiratory syndrome caused by coronavirus 2 (SARS-CoV-2) was reported in December 2019 in Wuhan, it has become evident that coronavirus infection can prompt multiorgan involvement [1]. In children and adolescents, SARS-CoV-2 is mainly linked to mild respiratory symptoms compared to the more severe forms seen in adults [2]. Although it has been reported that children and adolescents with COVID-19 disease have not required medical intervention, previously healthy children with SARS-CoV-2 infection have presented with symptoms of cardiovascular shock, hyperinflammation, and fever [3]. Recent data have shown an association between COVID-19 and vasculitis, which is considered a consequence of post-viral immunological reactions [4]. Moreover, many authors underline the increased cases of Kawasaki-like multi-inflammatory syndrome following SARS-CoV-2 infection [5]. These novel implications prompted the Centers for Disease Control and Prevention (CDC) to issue a national health advisory on May 14, 2020, which established diagnostic criteria for multiple inflammatory syndrome in children (MIS-C) associated with coronavirus disease 2019 [6]. Since SARS-CoV-2-induced vasculitis can be a life-threatening condition, it should always be considered in children previously diagnosed with Kawasaki disease (KD).

Given the growing number of multi-inflammatory system complications that have arisen during the SARS-CoV-2 pandemic, distinguishing between KD, MIS-C, and SARS-CoV-2 infection should prompt additional clinical attention. Assessing the immunological, serological, and epidemiological differences in KD patients with confirmed SARS-CoV-2 infection can help to estimate the risk of MIS-C development. This review reports the epidemiology, genetic predispositions, pathophysiology, clinical presentations of KD and MIS-C, as well as possible treatment options. It also underlines the necessity of future studies to document new and more effective treatment strategies in patients infected with SARS-CoV-2 and chart long-term outcomes more precisely.

## Methods

Medline and Pubmed were used to search for literature published during the last 10 years. Epidemiological, case–control and observational studies concerning Kawasaki disease and SARS-CoV-2 infections were analyzed in the context of MIS-C development. A comprehensive review of available data was used to describe the background of COVID-19 complications in three groups of pediatric patients affected by SARS-CoV-2 infection, KD, or MIS-C. The used keywords included Kawasaki disease, MIS-C, SARS-CoV-2, and COVID-19 with a combination of the following group of keywords: symptoms, clinical manifestations, epidemiology, etiology, pathogenesis, diagnosis, prognosis, and treatment.

### Epidemiology and demographics

#### SARS-CoV-2 infection

Epidemiological data regarding COVID-19 infection in the pediatric Chinese population has shown that children of all ages were prone to SARS-CoV-2 infection without apparent sex differences [7]. These data were further analyzed by Bhuiyan et al. who studied COVID-19 infection rates in children under 5 years of age. The meta-analysis showed that 53% of laboratory-confirmed COVID-19 patients were males while 47% were females, indicating no significant gender differences [8].

Although the SARS-CoV-2 virus can infect anyone regardless of race, some patients are more prone to severe infection than others. A cohort study by Moreira et al. demonstrated that African American children infected with COVID were at the highest risk for hospitalization compared to other races. The authors suggested that socioeconomic disparities could partly explain these epidemiological differences [9].

#### Kawasaki disease

KD is present predominantly in infants and young children. Nearly 80% of patients are below five years of age [10]. In younger patients, KD predominates in males in an approximately 1.5:1 male:female ratio [11]. The prevalence of this vasculitis is the highest in Asian populations and it has been well documented in Japan’s population [12]; however, it can develop in children of various descent, such as the United States (especially Hawaii), South Korea, and Taiwan [13, 14].

What is notable is the drop in incidence of KD in the COVID-19 era, particularly in countries where KD rates were highest before the pandemic, such as Japan, where most KD cases are reported. Lio et al. in a retrospective cohort study conducted in Kobe (a seaside city in Japan), demonstrated that KD incidence in children aged 0–4 decreased by 53% in 2020 compared to the average incidence in this age group from 2016 to 2019. Furthermore, the beginning of the decline of the incidence of KD in this population coincided with a COVID-19 outbreak in Kobe [15]. Similar findings were reported in South Korea. Before the pandemic, the annual mean incidence of KD in South Korea, which boasts the second-highest incidence rate worldwide, was 48.1 per 100,000 persons. However, after the onset of the pandemic and the implementation of nonpharmaceutical interventions (NPIs), such as mandatory mask-wearing, school closures, and testing and isolation of symptomatic individuals, this number diminished to 18.8 per 100,000 persons. The most remarkable change occurred in children between 0 and 4 years of age, the age group most commonly afflicted by KD. Before February 2020, the mean incidence rate in this cohort was 123.0/100000; however, between February and September 2020, that number dropped to 80.0/100000 [16].

Epidemiological analysis can be challenging. Even though some patients may fulfill full or partial criteria for KD, they will be designated as having MIS-C if they meet the case definition for MIS-C [6]. Thus, the incidence of KD after COVID-19 infection cannot be accurately documented.

#### Multiple inflammatory syndrome in children

MIS-C primarily affects school-aged children [17]. Feldstein et al. reported that the median age of development of MIS-C is about 8.3 years old [18]. Like in KD, males are more likely to develop MIS-C [19]. Thus, even though age is not a pathognomonic diagnostic factor, it can help to differentiate both conditions.

The overall incidence of MIS-C in the general population is 316 per one million SARS-Cov-2 infections (about one per 3000) in patients below 21 years old, including children [20]. Race seems to play a role in determining populations with a high risk of susceptibility to MIS-C development. Non-Hispanic Black and Hispanic children in the United States were found to be at a higher risk, while non-Hispanic White children developed MIS-C at rates lower than expected [19]. In a cross-sectional study published by Belay et al. in 2021, most patients diagnosed with MIS-C were either Hispanic or non-Hispanic Black [21]. However, Godfred-Cato et al. suggested that these differences may stem from inequalities in the social predictors of health, which can include housing, insurance, and finances. Furthermore, such socioeconomic factors have placed minority populations in the United States at higher risk for developing severe COVID-19 infection, of which MIS-C can be a complication [22].

### Pathophysiology and biomarkers

#### SARS-CoV-2 infection

In children hospitalized due to COVID-19, adequate immune system adaptation is related to host–pathogen responses. In hospitalized children, elevated anti-SARS-CoV-2 IgG but normal IgM level responses to the trimeric S glycoprotein on SARS-CoV-2 may be related to the more mild nature of COVID-19 disease in children. IgM, IgG and IgA recognize similar protein antigenic sites across the SARS-CoV-2 proteome, suggesting that SARS-CoV-2 infection induced a shared epitope profile. On the other hand, serum IgM antibodies from severe COVID-19 and severe MIS-C patients resulted in an approximately tenfold higher number of phage clones than IgG or IgA phage titers in the same patient cohort that presented with mild disease [23].

#### Kawasaki disease

In various connective tissue diseases, severe inflammation and multiple organ damage have been linked to previous infections. A similar mechanism is also observed in vasculitis, and it is further suspected that previous respiratory infections may trigger KD development [24]. In KD, the imbalance of chemical mediators from T-helper 17 and regulatory T-cell populations leads to hyper-inflammation [25]. The inflammation is also related to increased IgA plasma cells and macrophage infiltration in the bronchial epithelium. Rowley et al. used synthetic IgA that detected cytoplasmic antigen in the upper respiratory tract epithelium, suggesting that SARS-CoV-2 replicates in the bronchial epithelium and stimulates IgA response. Thus, this mechanism can partially explain the pathogenesis of KD [23].

The diagnosis of KD is based on clinical symptoms; however, some biochemical markers may be helpful in the diagnostic process of this vasculitis (e.g. elevated liver function tests, C-reactive protein, and erythrocyte sedimentation rate, leukocytosis, thrombocytosis, normocytic anemia and leukocyturia) [26]. One of the suggested serological factors in MIS-C is IL-21, produced by CD4+ and natural killer T cells (NKT) [27]. A high serum concentration of IL-21 is suggested as a serological KD marker, which supports a differential diagnosis between KD and mononucleosis. An elevated IL-21 level was found in Korean patients with KD and prolonged fever [28]. However, such relations were not observed in North American children [29].

The proinflammatory cytokines and specific antigens have also been detected in respiratory tracts in KD, which suggests that SARS-CoV-2 has a tropism for both vascular and respiratory cells [23]. In the first 2 weeks of KD, neutrophil infiltration occurs in the arterial walls, resulting in aneurysm formation [30]. Later, CD8+ T-cells, plasma cells, and monocytes infiltrate tissue and release proinflammatory cytokines, specifically IL-1β, and TNF-α, which may be present in cells for months to years [31]. During the chronic phase of the disease, myofibroblasts in the arterial walls can lead to vascular lumen stenosis. Moreover, in SARS-CoV-2 infection, the virus binds to ACE2 in the endothelial cells, resulting in hypercoagulability due to macrophage production of tissue factor via the nuclear factor(NF)-κB pathway [30]. These mechanisms in KD patients with SARS-CoV-2 infection provide premises for implementing antithrombotic treatment to the standard viral therapy.

#### Multiple inflammatory syndrome in children

A biochemical marker enabling MIS-C diagnosis in KD children following SARS-CoV-2 infection is a lack of lymphopenia and less significant thrombocytopenia, which progresses to thrombocytosis within the following 10–14 days. Conversely, subjects without KD who developed MIS-C are characterized by lymphopenia, low platelet counts, and low albumin levels [32]. Other markers include increased serum S100 protein and IL-18 concentration in patients with KD and MIS-C following SARS-CoV-2 infection, suggesting innate immune effectors' involvement. Moreover, IgG antibodies against S protein (spike protein) are an essential diagnostic factor for MIS-C [11]. They are stimulated by IFNγ-induced chemokine (C-X-C motif) ligand 9 (CXCL9) [33]. Interestingly, patients with MIS-C and lower CXCL9 concentrations clinically resemble KD patients, and they have a similar prevalence of coronary artery involvement.

**Fig. 1:**
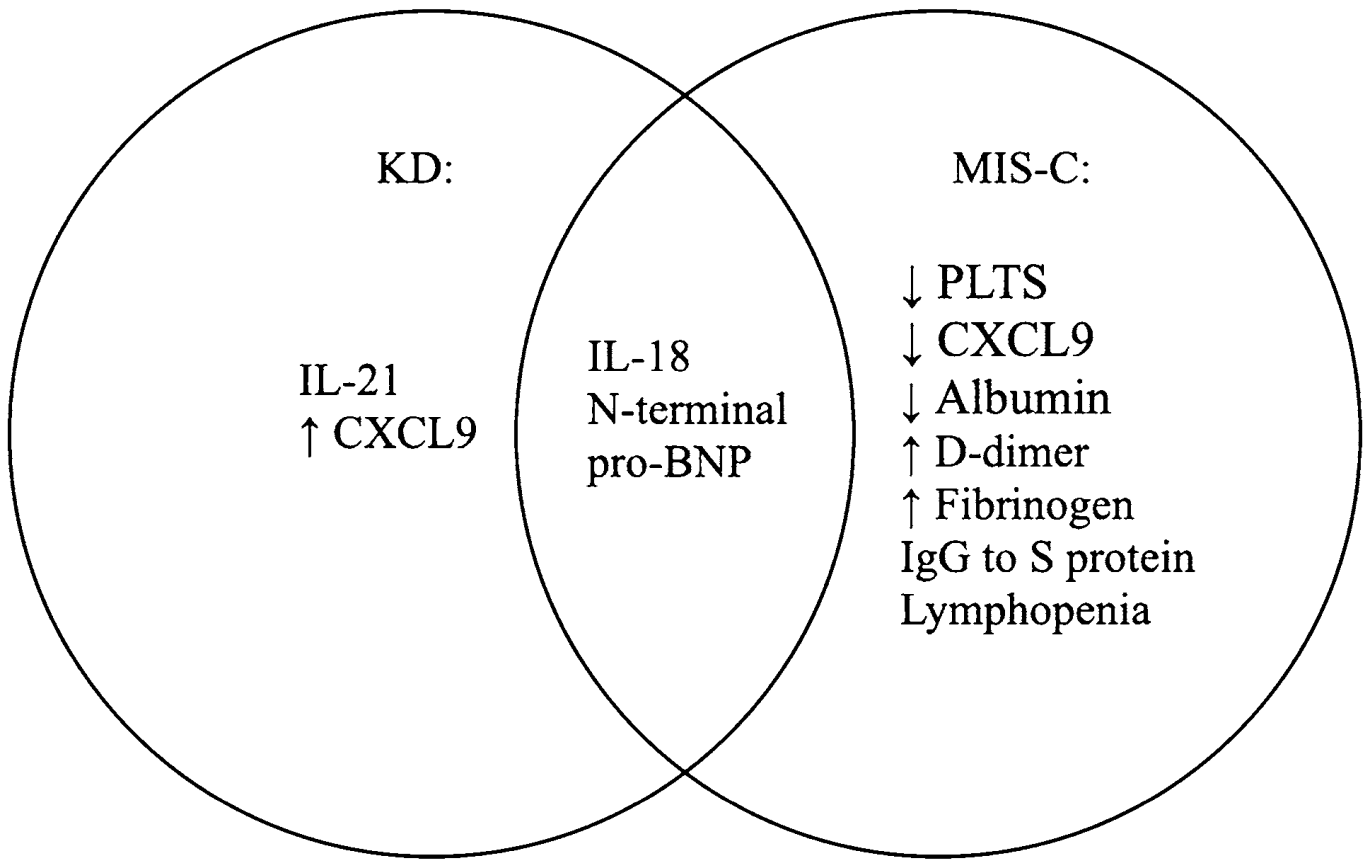
Diagnostic biomarkers present in KD, MIS-C or both. *KD* Kawasaki disease, *MIS-C* multisystem inflammatory syndrome in children, *IL-21* interleukin 21, *IL-18* interleukin 18, *PLTS* platelets, *CXCL9* chemokine (C-X-C motif) ligand 9, *pro-BNP* pro-B-type natriuretic peptide, *IgG* immunoglobulin G

There are differences in the MIS-C course associated with KD compared to the one MIS-C without KD. Loomba et al. demonstrated that the severe inflammatory response seen in COVID-19 triggered KD children might result from macrophage activation syndrome [MAS] [34]. One of the crucial inflammatory markers in MAS is ferritin, whose elevation results from histiocytic hyperactivity. Moreover, N-terminal pro-BNP, a potential biomarker of KD, is reported to be elevated up to 7000 pg/mL, increasing much more in MIS-C [35]. Carter et al. highlighted the significantly higher fibrinogen and d-dimer levels associated with decreased platelet amounts, suggesting a procoagulant state in the acute phase of MIS-C, absent in the acute phase of KD (Fig. 1). Nevertheless, both KD and MIS-C are characterized by a prothrombotic state, which further exacerbates in the setting of endothelial injury, immobilization, ventricular dysfunction, and coronary artery aneurysm development [36]. Other risk factors which may increase the prothrombotic state in MIS-C include age over 12 years old, comorbid malignancy, and the necessity of central venous catheters [37].

SARS-CoV-2-induced MIS-C is confirmed by epidemiological studies that show a higher prevalence of MIS-C cases in areas with the greatest incidence of SARS-CoV-2 infections [3]. Early invasion of the virus can trigger a cascade of macrophage activation with subsequent T-helper cell stimulation and cytokine synthesis (e.g. IL-1β, TNF-α) IL-4, IL-6, IL-12, IL-23, INF-ɣ). In turn, released cytokines enhance the activation of macrophages, neutrophils, and monocytes and stimulate plasma B cells, which produce autoantibodies against gastrointestinal, immune, and endothelial cells. Consequently, hyperimmune response leads to multisystem organ damage [38].

A novel marker of cytokine storm classically present in KD is IL-17; however, this interleukin is absent in children with MIS-C but elevated in KD patients who develop MIS-C [39]. Moreover, children with MIS-C were found to have a higher proinflammatory marker–TNF-α. This may result from resolved viral infection or decreased antiviral cytokines due to reduced viral load. MIS-C in children is not related to IL-1β elevation, which questions the current biological therapy in these patients [40]. Given these recent investigations, immunoprofiling of patients with KD, COVID-19 and MIS-C enables optimization of treatment strategies in KD and MIS-C. Due to the lack of studies involving children with previous KD diagnoses who developed MIS-C following COVID-19, this area requires future analysis and identification of specific diagnostic and prognostic markers. In the light of various molecular mechanisms of KD and MIS-C, recommendations for appropriate immunotherapies in specific clinical situations are needed.

### Genetic background

Analysis of the pathophysiology of KD and MIS-C and manifestations resulting from SARS-CoV-2 infection showed some genetic predispositions, which affect the regulation of immune system complex clearance and cellular adaptations.

#### Genetic implications of SARS-CoV-2 infection

An individual’s genetic background is important in predicting the clinical consequences of SARS-CoV-2 infection (Table 1). One of them is the polymorphism of the angiotensin system, which determines enzyme activity. The virus uses the angiotensin-converting enzyme (ACE) 2 receptor to enter the cell [41]. Moreover, an ACE deletion (D) polymorphism, which removes a 287 base pair marker in intron 16 on chromosome 17 in the *Angiotensin-Converting Enzyme* gene, may be responsible for the severity of SARS-CoV-2 infection in selected ethnic groups, particularly of African descent. Even though the deletion occurs in a non-coding region of the genome, its absence still carries downstream effects. After the virus binds to the ACE2 receptor, the receptor becomes downregulated. As a compensatory mechanism, the renin-angiotensin system (RAS) is excessively stimulated, contributing to the end-organ dysfunction that is a hallmark of severe SARS-CoV-2 infection. Interestingly, the ACE deletion (D) polymorphism is most commonly found in individuals of African descent and non-Hispanic Blacks in the USA. Consequently, increased morbidity and mortality in these ethnic groups infected by SARS-CoV-2 are observed [42]. In summary, the ACE deletion polymorphism genetic predisposition is defined as an inborn error of metabolism and is more prominent within certain ethnic groups. This polymorphism deletion plays a key role in the severity of SARS-CoV-2 infection due to the upregulation of RAS, which contributes to end-organ dysfunction.

**Table 1:** Genetic background and serological parameters in SARS-CoV-2

Diagnostic parameters useful in prognosis and treatment of SARS-CoV-2 infection		
Diagnostic sensitive and specific serotargets	Prognostic serological markers with associated disease severity	Therapeutic targets and cytokine signatures for immunotherapy
ORF8	COVID-19: ↑ORF5*	Spike fusion peptide
ORF3a	MIS-C: ↑ORF9c*	S2 HR2
NSP12	COVID-19: ↑IL-8	IL-1β inhibition
	MIS-C: ↑TNF	IL-6 inhibition

*ORF* open reading frame, *NSP* nonstructural protein, *IL-6* interleukin 6, *IL-8* interleukin 8, *TNF* tumor necrosis factor

*Higher affinity correlates with decreased disease severity

Following gene transcription and translation, RF5 (reading frame 5), ORF9c, ORF8, and ORF3a (open reading frame proteins), accessory protein antibody targets, are produced in response to SARS-CoV-2 infection. NSP12, an RNA polymerase implicated in Coronavirus replication, and S2 (spike protein 2) is a protein domain embedded within the HR2 (heptad region 2) of the spike protein of Coronavirus are also involved in the pathogenesis of COVID-19 [43]. The prognostic serological profile recognized with associated disease severity in Table 1 represents an IgM, IgA and IgG antibody epitope repertoire across the whole proteome of SARS-CoV-2.

Given new research, viral pandemic signatures (ViPs) have been shown to be implicated in KD, MIS-C, and COVID-19. These genes represent the host response to viral infection. These ViP signatures are induced in acute KD and may track disease severity, i.e. risk of developing giant coronary artery aneurysms (CAAs). ViPs have likewise been expressed in the setting of MIS-C, where their levels correlated with myocardial dysfunction. About 20-gene ViP signatures capture a core set of genes that are expressed in the setting of an overzealous (prolonged, intense or both) host immune responses in all three diseases: KD, COVID-19, and MIS-C. The overlapping ViP signatures found in KD, COVID-19, and MIS-C suggest that these three disease processes are clinically distinct states but can be placed along the same host immune response continuum. Taken together, these findings demonstrate that KD and MIS-C are fundamentally similar at the molecular level and that they share not only a common clinical presentation but also perhaps a proximal pathway of immunopathogenesis [44].

#### Genetic predisposition in Kawasaki disease

Genetic background is particularly underlined in first-degree relatives. For example, siblings of patients with KD have a nearly 30-fold increased risk of development of this vasculitis in comparison to the healthy population [14]. This suggests that the disease is not inherited in a Mendelian fashion [12]. However, recent studies underline the importance of specific polymorphisms in KD predisposition, despite not being inherited in a clear-cut way. One of them is the genetic polymorphism of the *ITPKC gene* (inositol-triphosphate three kinase C) [45]. The ITPKC protein is an isoenzyme that facilitates the phosphorylation of inositol 1,4,5-triphosphate (IP3) to inositol 1,3,4,5-tetraphosphate (IP4). As a result, it modulates calcium ion response to extracellular signals [46]. In addition, the *ITPKC* gene is a negative regulator of T-cell activation [47].

The *ITPKC* gene polymorphism is a result of C to G substitution in an intron between the first two exons in the *ITPKC* gene [45]. Such a substitution alters splicing efficiency and is related to disease outcome; for example, it is associated with increased T-cell activation, contributing to the pathogenesis of KD [48]. Onouchi et al. postulated that decreased levels of *ITPKC* mRNA result in an increased number of proinflammatory T-cells, which causes greater severity of KD. The C allele of *ITPKC* gene can reduce the splicing efficiency, resulting in hyperactivation of calcium-dependent nuclear factor of activated T-cells (NFAT) pathways in T-cells, which are essential in the immune response. Thus, this allele may contribute to the immune hyperactivity response demonstrated in KD patients [48]. Moreover, patients with the discussed polymorphism exhibit resistance to intravenous immunoglobulin (IVIG) used in KD treatment [45].

#### Inborn errors of immunity and MIS-C

Although knowledge regarding the genetic background of MIS-C is limited, it has been proposed that inborn errors of immunity (IEIs) may underlie its pathogenesis in a subset of patients. SARS-CoV-2 infection can activate a clinically silent monogenic or digenic IEI, precipitating an inflammatory reaction and resulting in clinical symptoms [49]. However, these genetic mutations need to be accurately analyzed with regard to age, ancestry, and mode of inheritance. Thus, future studies are required to illuminate the role of genetic mechanisms in developing MIS-C following SARS-CoV-2 infection.

### Clinical symptoms

#### SARS-CoV-2 infection

SARS-CoV-2 infection in children can manifest with various symptoms. While most patients are asymptomatic or exhibit mild symptoms, severe SARS-CoV-2 infections have also been documented [11]. A mild to moderate clinical course is characterized by nonspecific symptoms like fever, cough, myalgia and fatigue (Table 2). In contrast, a severe clinical development may result in dyspnea, hypoxia, central cyanosis, respiratory failure and multiorgan dysfunction [50].

**Table 2:** Clinical characteristics of KD, SARS-CoV-2 infection, and MIS-C

Characteristics of SARS-CoV-2-associated diseases			
Parameters analyzed	SARS-CoV-2 infection	KD	MIS-C
Pathology	Viral infection	Pediatric vasculitis	Hyperinflammatory syndrome
Mean age of disease onset	Any	< 5	6–11
Gender predisposition (M:F ratio)	1:1	1.5:1	1.5:1
Demographics	Worldwide	Japan	Hispanic and non-Hispanic Black in the USA
Genetic predisposition	ACE deletion (D) polymorphism	Genetic polymorphism of ITPKC	Inborn errors of immunity
Diagnostic procedure	PCR, antigen test, antibodies (reactive IgG or IgM to SARS-Cov-2)	No single specific diagnostic test	No single specific diagnostic test
Main diagnostic markers	Reactive IgG or IgM to SARS-CoV-2	IL-18, IL-21, Elevated N-terminal pro-BNP	IL-18, IgG against S protein, elevated N-terminal pro-BNP, lymphopenia
Similar symptoms	Fever, cough, fatigue	Fever (> 5 days if left untreated), lymphadenopathy, mucocutaneous findings, conjunctivitis, rash	Fever, lymphadenopathy, mucocutaneous symptoms, conjunctivitis, rash
Characteristic symptoms	Respiratory complications	Erythema and edema of extremities	Gastrointestinal findings
Pathognomonic symptoms	N/A	Strawberry tongue	N/A
Major complications	MIS-C	Coronary artery aneurysm	Myocardial dysfunction
Treatment	Often self-limited	IVIG, acetylsalicylic acid	IVIG, GCS

*SARS-CoV-2* severe acute respiratory syndrome coronavirus 2, *KD* Kawasaki disease, *MIS-C* multisystem inflammatory syndrome in children, *ITPKC* inositol-triphosphate three kinase C, *ACE* angiotensin converting enzyme, *PCR* polymerase chain reaction, *IL-18* interleukin 18, *IL-21* interleukin 21, *pro-BNP* pro-B-type natriuretic peptide, *IgG* immunoglobulin G, *IVIG* intravenous immunoglobulin, *GCS* glucocorticosteroids, *N/A* not applicable, *USA* United States of America

According to Sun et al., SARS-CoV-2 infection in pediatric patients can be classified as severe if the patient has an increased respiratory rate (≥ 30 times/min), has an oxygen saturation level < 93% under a resting state, or whose arterial pressure of oxygen (PaO_2_)/oxygen concentration (FiO_2_) ≤ 300 mm Hg. Critically ill patients develop respiratory failure requiring mechanical ventilation and have symptoms of septic shock or organ failure that necessitates ICU monitoring and treatment [51]. According to Sun et al., SARS-CoV-2 infection in pediatric patients can be classified as severe if the patient has an increased respiratory rate (≥ 30 times/min), has an oxygen saturation level < 93% under a resting state, or whose arterial pressure of oxygen (PaO2)/oxygen concentration (FiO2) ≤ 300 mm Hg. Critically ill patients develop respiratory failure requiring mechanical ventilation and have a septic shock or organ failure symptoms that necessitate ICU monitoring and treatment [51].

Dong et al. suggested that infants under the age of one were most susceptible to developing severe symptoms and more likely to progress to a critical state [7]. The risk of developing severe symptoms diminishes with increased patient age. Moreover, children with an underlying medical condition, such as chronic lung disease or immune suppression, were found to have poorer clinical outcomes than patients without comorbidities [9]. Nonspecific symptoms for SARS-CoV-2 infection may include cough, fever, myalgias, headaches, dyspnea, sore throat, diarrhea, nausea/vomiting, anosmia, ageusia, rhinorrhea, nasal congestion, chills/rigor, fatigue, confusion, and chest pain or pressure [6, 26, 52].

#### Kawasaki disease

KD is observed in infants and young children and predominantly affects small and medium-sized vessels [53]. The disease is of unknown etiology and, in developed countries, is one of the leading causes of acquired heart disease in children [26]. The first initial symptoms of infection include a high fever (lasting at least 5 days), mucocutaneous inflammation, and cervical lymph enlargement. Without treatment, the disease progresses and involves the coronary arteries, increasing the risk of coronary artery aneurysms [10]. Oral involvement classically presents as cracked lips with a strawberry tongue. Cutaneous involvement usually manifests as a desquamating rash of the hands and feet [12]. Other symptoms include bilateral conjunctival hyperemia, erythema, indurative palms, and feet edema [54].

#### Multiple inflammatory syndrome in children

A Kawasaki-like illness, presenting with similar clinical features, has been chronicled in previously healthy adolescent patients infected with or exposed to SARS-CoV-2. In symptomatic patients without confirmation of a positive diagnosis of SARS-CoV-2 (patient exposed to SARS-CoV-2 within one month of symptom onset), it is challenging to differentiate MIS-C from classic KD [11].

The CDC released guidelines in May 2020 outlining the definition of MIS-C. The diagnostic criteria include age < 21 years, fever, laboratory evidence of inflammation, and multisystem (at least two) organ involvement (cardiac, renal, respiratory, hematologic, gastrointestinal, dermatologic, or neurological). Multiorgan involvement is the indication for patient hospitalization. To diagnose the MIS-C a laboratory confirmation of SARS-CoV-2 infection is needed or documentation of SARS-CoV-2 exposure within four weeks of symptom onset in patients in whom an alternative diagnosis is not plausible [6]. MIS-C symptoms mimic KD manifestations, such as fever and mucocutaneous findings [11]. Unlike KD, however, gastrointestinal symptoms were reported in most patients with MIS-C [18]. Since there is considerable overlap between the signs, gender predisposition, and treatment options for both KD and MIS-C, both diseases should be carefully differentiated.

According to recommendations, children presenting with a low risk of MIS-C following COVID-19 infection should have inflammation screening, in contrast to patients diagnosed with MIS-C, who should be hospitalized [55]. Moreover, clinical observations based on the CDC's criteria for the signs and symptoms of MIS-C led to the classification of three subsets of patients. The first group is characterized by multiorgan involvement with prominent cardiovascular and gastrointestinal symptoms. The second group of patients presents mainly respiratory symptoms resembling COVID-19, while the third group exhibits a rash and mucocutaneous lesions, which coincidentally fulfill the diagnostic criteria for KD [56]. Therefore, it is crucial to distinguish patients based on their initial presenting signs and symptoms to provide appropriate treatment and post-hospitalization follow-up.

#### Diagnostic criteria of KD, MIS-C and SARS-CoV-2

Since various symptoms and course of MIS-C can be present, which may resemble KD diseases, the diagnosis of this syndrome can be challenging (Table 3). For example, MIS-C can develop later than it is pointed in diagnostic criteria (e.g. after 16 weeks after SARS-CoV2 exposure compared to typical MIS-C development within 2–6 weeks after viral contact) [43]. The biochemical and serological markers may help in the differential diagnosis of MIS-C in both previously healthy or SARS-CoV2 infected children (Fig. 1).

**Table 3:** Diagnostic criteria of KD, MIS-C and SARS-CoV-2

Diagnostic criteria of SARS-CoV-2 infection, KD, and MIS-C			
Analysed parameters	SARS-CoV-2	KD	MIS-C
Initial diagnosis	Positive NAAT test from upper respiratory tract OR negative NAAT test with supporting laboratory and clinical findings	Anytime	≅ 2–6 weeks post-SARS-CoV-2 infection
Fever	Not needed for diagnosis	Typical: unexplained for ≥ 4 days	> 38.0℃ for ≥ 3 days
		Atypical: unexplained for ≥ 5 days	
Clinical manifestations	Not specific for COVID-19	Typical: 4/5 findings*	At least 2 signs of multisystem involvement
		Atypical: 2–3/5 findings*	
Imaging method of choice	N/A	Transthoracic echocardiography	Transthoracic echocardiography
Evidence of current/recent SARS-CoV-2 infection	Yes	No	Yes
Laboratory markers	Reactive IgM or IgG to SARS-CoV-2	Evidence of inflammation (e.g. CRP)	Evidence of inflammation (e.g. CRP)
Age	Any	Usually < 5 years	< 20 years

*SARS-CoV-2* severe acute respiratory syndrome coronavirus 2, *KD* Kawasaki disease, *MIS-C* multisystem inflammatory syndrome in children, *COVID-19* coronavirus disease 2019, *CRP* C-reactive protein, *IgG* immunoglobulin G, *IgM* immunoglobulin M, *NAATs* nucleic acid amplification tests, *PT-PCR* reverse transcription-polymerase chain reaction

*Findings include bilateral bulbar conjunctival infection, oral mucous membrane changes, peripheral extremity changes (e.g. erythema of palms/soles, edema of hands/feet, periungual desquamation), polymorphous rash and/or cervical lymphadenopathy (at least 1 lymph node ≥ 1.5 cm in diameter)

While many clinical features of KD, MIS-C, and SARS-CoV-2 have overlapping manifestations, the diagnostic criteria are helpful in distinguishing analyzed diseases. An adequate diagnosis enables proper treatment and evaluation of patient prognosis. One of the diagnostic tests is NAATs (nucleic acid amplification tests), including PT-PCR (reverse transcription-polymerase chain reaction), transcription-mediated assay, or strand displacement assay.

### Treatment

#### SARS-CoV-2 infection

Most children infected with COVID-19 are treated with supportive care measures [53]. In severe cases, antiviral treatment medications may be considered. Remdesivir may be administered to critically ill children who require oxygen therapy. However, some drugs used to treat infected adults (e.g. azithromycin, protease inhibitors, and hydroxychloroquine) are discouraged in children [57]. Furthermore, administration of COVID-19 monoclonal antibodies is not recommended in the pediatric population [58].

#### Kawasaki disease

IVIG and acetylsalicylic acid are the proposed first-line therapies for managing KD. Treatment with IVIG has ameliorated the rash, persistent fever, and conjunctivitis that characterize KD [11]. In high-risk KD patients who are unresponsive to IVIG, the administration of glucocorticosteroids (GCS) is recommended to minimize the risk of coronary artery aneurysms. Other treatment possibilities include infliximab (a monoclonal antibody against TNF), cyclosporin (a calcineurin inhibitor), and anakinra (an IL-1 receptor antagonist) [52].

#### Multiple inflammatory syndrome in children

Although most pediatric cases of SAR-CoV-2 infection are self-limited, those that progress to MIS-C must be treated according to the current understanding of the underlying disease mechanism. Because MIS-C involves overactive proinflammatory immunologic mechanisms and consequent hyperinflammatory response, treatment approaches should modulate immune system activity. The American College of Rheumatology (ACR) recommends administering IVIG (at a dose of 2 gm/kg) as a first-line therapy dedicated to hospitalized patients with MIS-C. If organ dysfunction or septic shock develops, GCS (at a dose of 1–2 mg/kg/day) should be added [52].

For patients who do not respond to IVIG or steroids (or these medications are contraindicated), high doses of anakinra should be considered (> 4 mg/kg/day). Since left ventricular dysfunction can be a life-threatening complication of MIS-C, echocardiograms should be performed every day for 7–14 days and then 4–6 weeks after disease onset [26]. If left ventricular dysfunction is reported in the early acute phase of MIS-C, an echocardiogram should be repeated one year after MIS-C diagnosis [52]. Every patient recovering from MIS-C may be discharged from the hospital three to four days after evidence of decreased inflammatory markers (CRP, d-dimer, ferritin), diminished troponin level, absence of fever for 48 h, normal EKG, and stable findings on echocardiography [55].

## Conclusion

KD and MIS-C following SARS-CoV-2 infection are two pediatric conditions with similar clinical features, such as fever and mucocutaneous findings. However, some symptoms and biochemical markers are different in both pathologies, such as CXCL9, fibrinogen, d-dimer, albumin, platelets, and IL-21. Moreover, KD develops most often in young children in Japan and those with an *ITPKC* gene polymorphism, while MIS-C is most often identified in Hispanic or non-Hispanic Blacks and those with IEIs. Nevertheless, it is difficult to assess the risk of developing MIS-C in KD patients in whom a positive diagnosis of SARS-CoV-2 is confirmed. Future studies and long-term observations are needed to elucidate these two conditions' pathophysiologic and genetic background factors.

Additionally, strict monitoring is required for every SARS-CoV-2-infected patient with KD, since severe complications can develop. Due to lack of evidence and prolonged observations, which would otherwise enable the differentiation of KD with associated SARS-CoV-2 infection from MIS-C, studies should focus on assessing the genetic basis, immune profiles, and specific diagnostic markers to target therapeutic strategies better. These approaches would more efficiently and successfully manage complications that influence short- and long-term prognosis.

Long-term outcomes in KD patients who develop MIS-C due to SARS-CoV-2 infection have been inadequately documented due to the timing of the pandemic. While short-term consequences of SARS-CoV-2 infection in patients with KD have been outlined, future studies should aim for long-term observation of physiological adaptations as well as the onset of possible new symptoms. There is a need to estimate the impact of socioeconomic inequalities of SARS-CoV-2 patients on the development of MIS-C in KD patients. Future works should also suggest why males develop KD and MIS-C more frequently despite equal SARS-CoV-2 infection rates between both genders.
